# Sphingosine 1-Phosphate Receptor Modulator Fingolimod (FTY720) Attenuates Myocardial Fibrosis in Post-heterotopic Heart Transplantation

**DOI:** 10.3389/fphar.2017.00645

**Published:** 2017-09-15

**Authors:** Naseer Ahmed, Daniele Linardi, Nazeer Muhammad, Cristiano Chiamulera, Guido Fumagalli, Livio San Biagio, Mebratu A. Gebrie, Muhammad Aslam, Giovanni Battista Luciani, Giuseppe Faggian, Alessio Rungatscher

**Affiliations:** ^1^Section of Cardiac Surgery, Department of Surgery, Dentistry, Paediatrics and Gynaecology, University of Verona Verona, Italy; ^2^Faculty of Health Sciences, University of Punjab Lahore, Pakistan; ^3^Research Unit, Faculty of Allied Health Sciences, University of Lahore Lahore, Pakistan; ^4^Section of Pharmacology, Department of Diagnostics and Public Health, University of Verona Verona, Italy; ^5^COMSATS Institute of Information Technology Wah Cantt, Pakistan; ^6^Department of Anatomy, Università di Addis Abeba Addis Ababa, Ethiopia; ^7^Department of Internal Medicine, Cardiology and Angiology, University Hospital, Justus Liebig University Giessen, Germany

**Keywords:** sphingosine 1-phosphate, cardiac fibrosis, ischemia/reperfusion injury, cardioprotective, heart transplantation

## Abstract

**Background and Objective:** Sphingosine 1-phosphate (S1P), and S1P receptor modulator fingolimod have been suggested to play important cardioprotective role in animal models of myocardial ischemia/reperfusion injuries. To understand the cardioprotective function of S1P and its mechanism *in vivo*, we analyzed apoptotic, inflammatory biomarkers, and myocardial fibrosis in an *in vivo* heterotopic rat heart transplantation model.

**Methods:** Heterotopic heart transplantation is performed in 60 Sprague–Dawley (SD) rats (350–400 g). The heart transplant recipients (*n* = 60) are categorized into Group A (control) and Group B (fingolimod treated 1 mg/kg intravenous). At baseline with 24 h after heart transplantation, blood and myocardial tissue are collected for analysis of myocardial biomarkers, apoptosis, inflammatory markers, oxidative stress, and phosphorylation of Akt/Erk/STAT-3 signaling pathways. Myocardial fibrosis was investigated using Masson’s trichrome staining and L-hydroxyline.

**Results:** Fingolimod treatment activates both Reperfusion Injury Salvage Kinase (RISK) and Survivor Activating Factor Enhancement (SAFE) pathways as evident from activation of anti-apoptotic and anti-inflammatory pathways. Fingolimod treatment caused a reduction in myocardial oxidative stress and hence cardiomyocyte apoptosis resulting in a decrease in myocardial reperfusion injury. Moreover, a significant (*p* < 0.001) reduction in collagen staining and hydroxyproline content was observed in fingolimod treated animals 30 days after transplantation demonstrating a reduction in cardiac fibrosis.

**Conclusion:** S1P receptor activation with fingolimod activates anti-apoptotic and anti-inflammatory pathways, leading to improved myocardial salvage causing a reduction in cardiac fibrosis.

## Introduction

Heart transplantation is the ultimate treatment option for heart failure (HF) (Koerner et al., 2000), which is a major health problem worldwide, with a prevalence of 23 million worldwide and 5.8 million in the United States alone (Benjamin et al., 2017). HF has been singled out as an emerging epidemic in the world with higher morbidity and mortality in 65 years old people and elders (Roger, 2013). Cardiac fibrosis is one of the main factors in the development and progression of HF. Therefore, targeting the development and progression of cardiac fibrosis is a critical goal in the treatment of HF (Miner and Miller, 2006; Opie et al., 2006).

Myocardial fibrosis can be categorized in two distinct forms: (1) ischemia/reperfusion (I/R) injury as a result of myocardial infarction, cardiac arrest, cardioplegic arrest, and cardiac transplantation due to reparative process by replacement of cardiomyocytes with collagen matrix and (2) interstitial or perivascular fibrosis as a consequences of trauma caused by volume or pressure overload, hypertrophic, or dilated cardiomyopathies (Dobaczewski and Frangogiannis, 2009; Krenning et al., 2010). Most common cause of cardiac dysfunction and mortality in cardiac transplant patients is due to I/R injury (Murata et al., 2004; Tanaka et al., 2005). Currently, low potassium solutions give some positive evidence for better cardiac graft preservation and demonstrate attenuation of grafted heart damage related to cold storage and I/R injury (Rudd and Dobson, 2009). However, it has been observed that this solution is not sufficient to protect from myocardial damage, due to decreased cellular energy reserves, release of oxidants, myocardial stunning, and myocardial dysfunction. Till now, there is no efficient strategy that can reduce cardiac fibrosis due to heart procurement and transplantation.

Sphingosine 1-phosphate (S1P) is a bioactive lipid that is characterized by a peculiar mechanism of action. In fact S1P, which is produced intracellularly, can act as an intracellular mediator, whereas after its export outside the cell, it can act as ligand of specific G-protein coupled receptors, which were initially named endothelial differentiation gene (EDG) and eventually renamed sphingosine 1-phosphate receptors (S1PRs). Currently five S1P (S1P1-5) receptors are known. The S1P1, S1P2, and S1P3 receptors are ubiquitously expressed whereas the expression of S1P4 and S1P5 receptors is highly restricted to the immune and nervous system (Ishii et al., 2004; Chun et al., 2010). In cardiac tissue, S1P1 receptor is the predominant receptor, whereas the expression of S1P2 and S1P3 receptors is relatively very low (Zhang et al., 2007). Keul et al. (2016) demonstrated in experimental study that S1P1 receptor is vital for regulating cardiac function by modulating ion channels and mediates myocardial protection by ischemic preconditioning.

Sphingosine 1-phosphate receptor modulator fingolimod is a lipophilic agent derived from the fungus *Isaria sinclairii* (Chiba and Adachi, 2012). S1P may also take part in cardioprotection against I/R injury (Somers et al., 2012). S1PR modulator fingolimod has potent anti-inflammatory and anti-oxidant properties, by inhibiting oxygen free radical and leading to reduced myocardial fibrosis, as a consequence of less cardiomyocytes death (Aytan et al., 2016).

Fingolimod activates Reperfusion Injury Salvage Kinase (RISK) and Survivor Activating Factor Enhancement (SAFE) pathways, leading to inhibition of pro-apoptotic proteins and activation of anti-apoptotic proteins (Liu et al., 2013). We hypothesized that pre-conditioning with fingolimod might achieve optimal cardioprotection and reduce fibrosis by activating anti-apoptotic, anti-inflammatory, and pro-survival pathways. Our aim was to investigate the cardioprotective role of fingolimod on apoptosis, inflammation, oxidative stress, nitrative stress, activation of Akt and Erk1/2 signaling pathways, and cardiac fibrosis in rat heterotopic heart transplantation model.

## Materials and Methods

### Animals

Sprague–Dawley (SD) Rats (350–400 g) were obtained from Harlan Laboratories (Udine, Italy). The rats were fed standard rat chow with an access to *ad libitum*. The rats were housed at a density of 4–6 per cage and maintained on a 12 h light/dark cycle at 21°C. All the animal experiments were carried out according to the regulations (Declaration of Helsinki and “Guide for the Care and Use of Laboratory Animals” – Institute of Laboratory Animal Resources – National Institutes of Health) after experimental protocols were reviewed and approved by the Ethics Committee of University of Verona and the Italian Ministry of Health (341/2016-PR).

### Experimental Design

**FIGURE 1:**
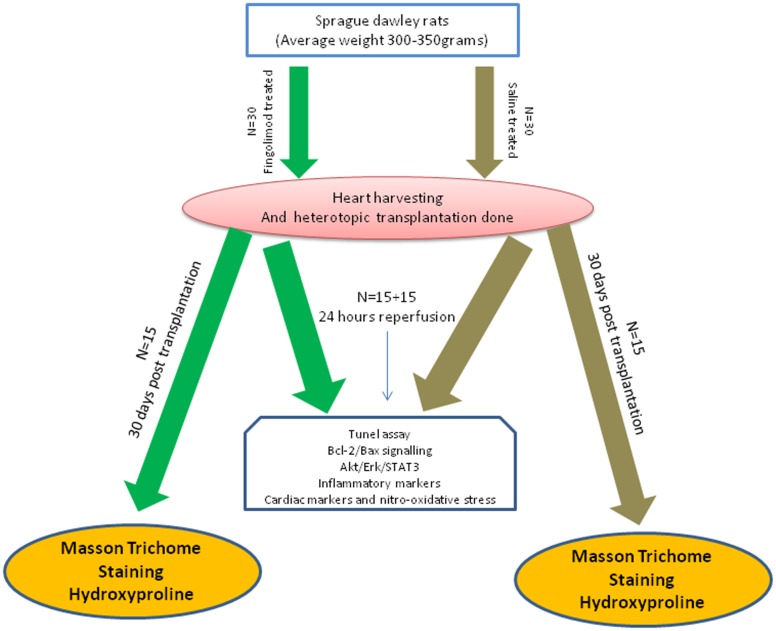
Schematic presentation of experimental design.

In this study, 120 animals were used, 60 donors and 60 recipients. The 60 recipient rats were divided into two main groups (control group and fingolimod treated group). The experimental protocol used was as follows: the animals were pre-treated 15 min before heart explantation, by intravenous (i.v.) injection of either saline (control group) or fingolimod [treated group, 1 mg/kg; (Santos-Gallego et al., 2016)]. Hearts were harvested and transplantation was performed within 1 h. Abdominal cavity was closed and animals were allowed to recover. Transplanted animals were then divided into two subgroups: first subgroup was sacrificed after 24 h of R and second group was sacrificed 30 days after transplant (*n* = 15 for each group). The 24 h group was used to collect blood aliquots and hearts for cardiac, inflammatory, apoptotic, and oxidative markers while 30 day group was used to study cardiac fibrosis using Masson’s trichrome staining and L-hydroxyproline. Schematic presentation of the experimental plan is illustrated in **Figure 1**.

#### Surgical Technique

Recipient rats were anesthetized with 5% isoflurane in 50% O_2_ administered through a facial mask. Median abdominal incision was practiced. Sub-renal aortic and inferior vena cava (IVC) are exposed and isolated. Two vascular clamps were positioned superiorly and inferiorly to the site of transplanted heart implantation. Two adequate incisions were performed on aorta and IVC and the lumen of the two vessels was perfused with saline solution and heparin to avoid thrombus formation.

While an operator sets up the recipient rat another operator prepares the donor rat. Anesthesia was performed by intraperitoneal administration of sodium thiopental (Pentotal^®^) 60 mg/kg. The animal was intubated orotracheally with an atraumatic tube consisting of a 16 G venous cannula and was mechanically ventilated (Harvard Model 687; Harvard Apparatus, Holliston, MA, United States). Tidal volume was set to 10 ml/kg and respiratory rate 60 breaths/min with an air–oxygen mixture/FiO_2_ = 0.5). Anesthesia was maintained with isoflurane (2%) during the whole procedure.

The chest was opened through median sternotomy and heart was exposed. The right superior vena cava (SVC) was tied with Ticron 3.0 suture, ascending aorta and pulmonary artery are resected at 3–4 mm from their origin with an angled scissor.

Ten milliliters of cold S. Thomas cardioplegic solution was injected to stop the heartbeat, and after that IVC was tied and closed with Ticron 3.0 suture. The heart was then lifted up and with a single ligature pulmonary veins and left SVC were tied and closed. The heart was immediately dipped in a cold cardioplegia bath and another 10 ml of S. Thomas solution was injected into the heart through ascending aorta.

The explanted heart was then placed in the abdomen of the recipient rat and positioned in order to avoid twisting. The first anastomosis was performed between the abdominal aorta of the recipient rat and the ascending aorta of the donor heart using polypropylene 8.0 continuous suture. After completing this anastomosis, the anastomosis between IVC of the recipient rat and the pulmonary artery of the donor heart was performed. The heart was topically cooled by irrigating intermittently with saline solution at 4°C during the transplantation period. Time for the execution of the two anastomoses was between 35 and 40 min. Afterward, the vascular clamps were removed and if no stenosis or bleeding is evident, the implanted heart spontaneously starts beating. Warm saline solution was poured into the abdominal cavity and the abdomen was closed through continuous suture of the muscle wall and of the skin with a Polypropylene 4.0 suture. Sedation was suspended and after few minutes rat wakes up.

### TUNEL Assay

Paraffin-embedded hearts from control and treated animals were deparaffinised with xylene and gradually hydrated in ethanol (EtOH) solutions (100, 95, 70, and 50%). Apoptotic cells were stained with terminal deoxynucleotidyl transferase dUTP nick end labeling (TUNEL) assay using the *In Situ* Cell Death Detection Kit (Roche, Cat. 11684809910). Nuclei were stained with Hoechst 33342 solution and quantified using ImageJ software (United States National Institutes of Health).

### Histology and Myocardial Fibrosis Quantification

Myocardial tissues from the left ventricle were fixed in 10% formaldehyde in phosphate-buffered saline (PBS) (pH 7.2), after fixation these blocks were embedded in paraffin and 3 μm thick sections were prepared for histological staining (Song et al., 2014). Myocyte diameter was determined from haematoxylin–eosin stained ventricle sections and expressed in micrometer. Cardiac fibrosis was analyzed from tissue sections stained with Masson’s trichrome staining.

### L-Hydroxyproline Assay

The L-hydroxyproline content is a type of protein that indicates collagen deposition. Using the L-hydroxyproline assay, collagen deposition was evaluated to assess tissue damage. Cardiac tissue was minced and hydrolyzed with HCl at 125°C and 200 psi for 2 h. After serial evaporation and addition of water final product was reconstituted in 5 ml distilled water. Each sample (2-ml aliquot) was oxidized with 1 ml of chloramine-T for 20 min. The remaining chloramine-T was neutralized with 1 ml of perchloric acid (3.0 M). The sample was mixed with 1 ml of *p*-dimethylaminobenzaldehyde and incubated in a hot water bath at 60°C and cooled to room temperature (RT) afterward. The absorbance was measured at 557 nm and the L-hydroxyproline concentration was measured using a standard curve.

### Western Blotting

The heart tissue was homogenized in buffer containing 1% Triton X-100 with phosphatase and protease inhibitors cocktail (Sigma–Aldrich, Milan, Italy) as described previously (Giani et al., 2007). The tissue extracts were centrifuged at 16,000 × *g* for 15 min at 4°C to remove the insoluble material, and supernatants were collected in separate aliquots. Protein concentration was measured by using the BCA Assay Kit (Beyotime Institute of Biotechnology, China) according to manufacturer’s instruction. The phosphorylation levels of Akt1/2 and ERK1/2 equal amounts of solubilized proteins (35 μg) were denatured by boiling for 5 min at 100°C in reducing buffer, resolved by SDS-PAGE, and protein transferred to polyvinylidene difluoride (PVDF) membranes. After transfer, membranes were blocked in blocking solution (5% BSA in TBS-T) for 1 h. The membranes were then incubated with primary antibodies (1:1000) overnight at 4°C. The membranes were washed with TBS-T and incubated with HRP-conjugated secondary antibodies (1:10,000). The bands were visualized by using Syngene Western Blotting detection system (Syngene, Cambridge, United Kingdom). The quantification of protein band densities was performed by ImageJ software (United States National Institutes of Health). GAPDH was used as loading control. The antibodies used were: total Akt1/2 (Abcam, Cambridge, United Kingdom), anti-phosphorylated Akt1/2 (rabbit, Cell Signaling), total ERK1/2 (Abcam, Cambridge, United Kingdom), anti-phosphorylated ERK1/2 (Cell Signaling, Denver, CO, United States), anti-STAT3 (Cell signaling, Denver, CO, United States), and HRP-conjugated goat anti-rabbit IgG (Abcam, Cambridge, United Kingdom). The remaining reagents were purchased from Sigma–Aldrich (St. Louis, MO, United States).

### Immunohistochemical Staining

Paraffin-embedded left ventricle sections were heated at 60°C for 1 h and rehydrated with xylene for 20 min and with graded EtOH solutions (100, 95, 70, and 50%). Antigen retrieval solution was applied (0.01 M citrate buffer, pH 6.0) for 30 min in a boiling water bath, followed by slow cooling and rinsing with PBS. Endogenous peroxidase activity was suppressed by incubation with 3% hydrogen peroxide in TBS-T. Myocardial tissue sections were incubated with primary antibodies Bax (Abcam, Cambridge, United Kingdom) and Bcl-2 (Dako, Glostrup, Denmark) were incubated overnight at 4°C. Afterward, the sections were incubated with HRP-conjugated secondary antibody for 30 min, and diaminobenzidine and hydrogen peroxide chromogen substrate (DAB, Dako Corp.). All sections were counterstained with haematoxylin and mounted. The negative controls were incubated with non-immune rabbit IgG instead of primary antibody. All histological sections were studied using a light microscope Nikon E400 (Nikon Instrument Group, Melville, NY, United States).

### Detection of Cardiac Enzymes and Oxidative Stress

After transplantation and R, blood was taken from the carotid artery and was placed at RT for 30 min to allow coagulation. The serum was then collected by centrifugation and placed at -70°C for preservation. Serum CK-MB and cardiac troponin-I were measured using CK-MB and Cardiac Troponin-I Assay Kits (Sigma–Aldrich, United Kingdom) and Oxidative Stress Kit (Thermo Fisher Scientific, United States) according to manufacturer’s instruction.

### Statistical Analysis

To compare treatment and control groups, all measurements and results are presented as mean ± SEM. Both groups were compared using Student’s *t*-test or Mann–Whitney *U* nonparametric test. A *p*-value of less than 0.05 was considered to be statistically significant. Analyses were performed using SPSS software version 21 (SPSS Inc., Chicago, IL, United States).

## Results

### Expression of Bcl-2 and Bax

**FIGURE 2:**
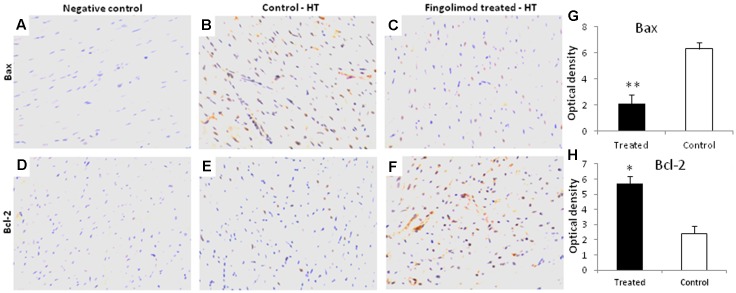
Representative images (*n* = 15 for each group control and treated group) of myocardial tissue expression levels of Bcl-2 and Bax after 60 min of ischemia and 24 h reperfusion in fingolimod treated and control group. The protein expression levels of Bcl-2 and Bax were determined by an immunohistochemistry. **(A,D)** Negative control, **(B,E)** Bax and Bcl-2 expression in HT-control group, respectively, **(C,F)** Bax and Bcl-2 expression in HT-fingolimod treated group, **(G,H)** graphical presentation of Bax and Bcl-2 protein in fingolimod treated and control group. Data presented as a mean ± SEM. *P*-value < 0.05 considered as significant. (^∗^*p* < 0.05 and ^∗∗^*p* < 0.001, treated vs. control).

Myocyte apoptosis is hallmark of reperfusion injury after HT. Therefore, the expression of pro-apoptotic Bax and anti-apoptotic Bcl-2 was analyzed in our HT model. As presented in **Figure 2**, after 24 h of reperfusion there was a massive expression of Bax in control group (**Figure 2B**) which was abolished by S1PR agonist fingolimod (**Figures 2C,G**). Likewise, the expression of anti-apoptotic Bcl-2 was significantly enhanced in fingolimod treated group (**Figures 2E,F,H**).

### Effect of Fingolimod on Apoptosis

**FIGURE 3:**
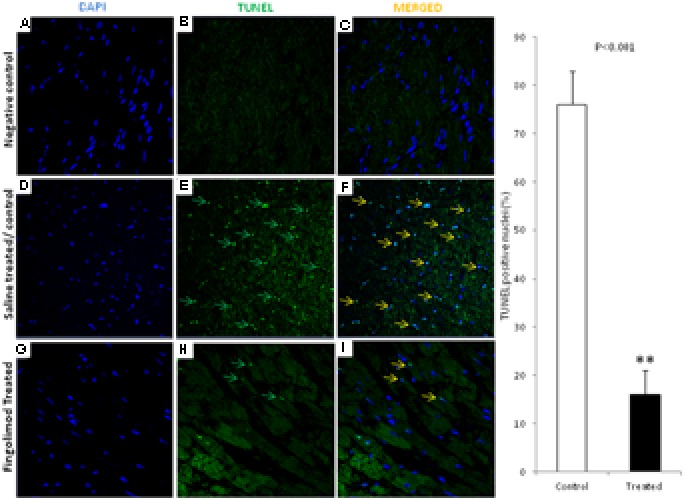
Representative images (*n* = 15 for each group control and treated group) of immunofluorescent staining for TUNEL-positive nuclei in negative control, HT-control, HT-fingolimod treated groups. **(A,D,G)** Shows only DAPI in myocardial tissue, **(B,E,H)** TUNEL signals, and **(C,F,I)** merged images. TUNEL-positive myocytes were much lower in numbers frequently in control HT group than in HT-fingolimod treated group. Green and yellow arrows indicate apoptotic nuclei and apoptotic nuclei merged with DAPI, respectively. Original magnification 40×. Data presented as a mean ± SEM. *P*-value < 0.05 considered as significant. (^∗∗^*p* < 0.001, treated vs. control).

Myocyte apoptosis was analyzed by TUNEL assay. As shown in **Figures 3D,E,F** massive apoptosis was observed in the control group after 24 h of transplantation compared to healthy non-operated hearts (**Figures 3A–C**). This was significantly attenuated in fingolimod group (**Figures 3G,H,I**).

### Collagen Deposition and Cardiac Fibrosis

**FIGURE 4:**
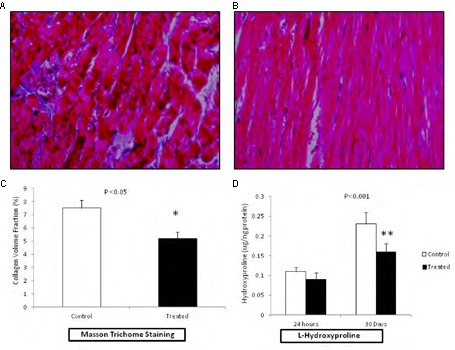
Representative photomicrograph (*n* = 15 for each group control and treated group) showing the collagen deposition in rats related to HT I/R injury (20× magnification). Fingolimod decreased post-HT myocardial in myocardial fibrosis. The viable myocardium is stained bright red. Fibrosis is stained bright blue. **(A)**; HT control after 30 days of reperfusion and **(B)**; HT fingolimod treated after 30 days of reperfusion. **(C)** Graphical presentation of cardiac fibrosis, and **(D)** graphical presentation of L-hydroxyproline at early and late phase of R injury related to transplantation. Data presented as a mean ± SEM. *P*-value < 0.05 considered as significant (^∗^*p* < 0.05 and ^∗∗^*p* < 0.001, treated vs. control).

Development of cardiac fibrosis was analyzed by measuring the collagen deposition in the cardiac tissue after 30 days of the reperfusion. Collagen deposition was analyzed by Masson’s trichrome staining. Extensive collagen deposition in the ventricular tissue as indicated by blue staining was observed after 30 days of HT (**Figures 4A,B**). Interestingly, hearts previously perfused with fingolimod before transplantation were protected from fibrosis as indicated by much reduced collagen deposition (**Figures 4B,C**). L-Hydroxyproline assay has been performed to investigate collagen content in myocardial tissue post-cardiac transplantation at early and late phase of reperfusion (**Figure 4D**). This quantitative assay confirmed our histological findings that showed reduction of cardiac fibrosis with treatment of fingolimod prior to heart transplantation.

### Serum Levels of Inflammatory Mediators

**FIGURE 5:**
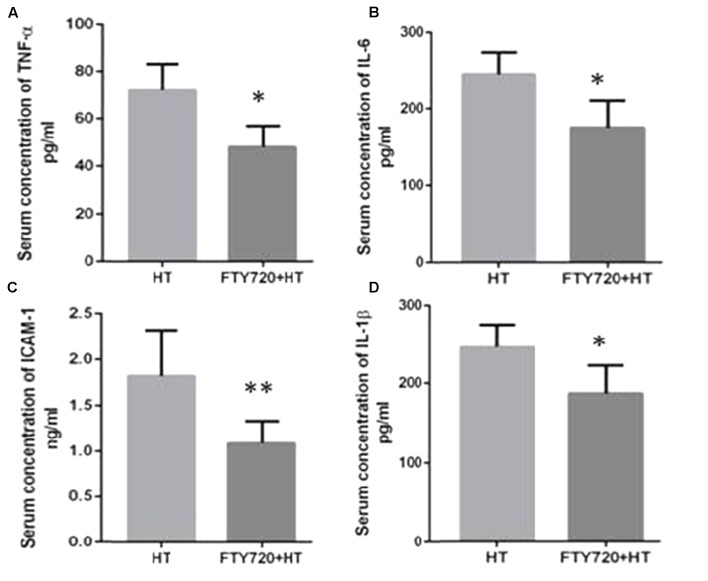
Myocardial production of TNF-α **(A)**, IL-6 **(B),** ICAM-1 **(C)**, and IL-1β **(D)** after 24 h of R. **(A)** HT model without fingolimod treatment shows high expression of TNF-α as compared to fingolimod treatment. **(B)** HT-R induced significant high IL-6 after 24 h of R compared with the fingolimod treated. **(C)** Fingolimod treated group remarkably reduced the production of the ICAM-1 release as compared to Control. **(D)** This section of the panel presents production of 1L-1β higher in control vs. fingolimod treated group in HT-R group. Each bar height represents the mean ± SEM (each group *n* = 15) (^##^*p* ≤ 0.01 vs. baseline, ^∗^*p* ≤ 0.05 and ^∗∗^*p* ≤ 0.001 vs. HT-control group).

The inflammatory mediator’s contribution in tissue injury has previously been identified in the heterotopic HT models (Mori et al., 2014). During heterotopic HT-related I/R injury a significant elevation in the plasma levels of cytokines mainly TNF-α, IL-1β, IL-6, and ICAM-1 may result (Vinten-Johansen et al., 2007). Like previous reports, increased serum levels of these mediators were observed in HT animals after 24 h of R (**Figure 5**) which were significantly attenuated in animals transplanted with hearts pre-treated with fingolimod.

### Effect of Fingolimod on Erk42/44, Akt1/2, and STAT3 Phosphorylation

**FIGURE 6:**
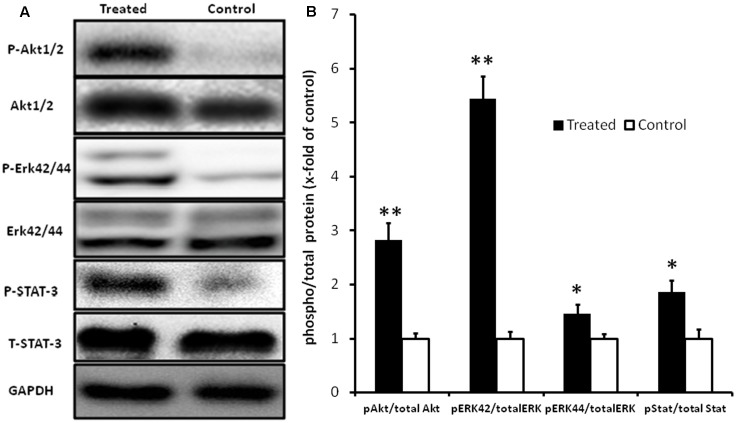
Representative Western blot images **(A)** and graphical presentation of the ratio of pAkt1/2/Akt, pErk42/44/Erk, and pSTAT-3/STAT-3 **(B)**. Myocardial samples were collected at the end of 24 h reperfusion. Activation of Akt1/2, Erk42/44, and STAT-3 pathways in fingolimod treated group was measured by change in fold of phosphorylation with respect to control group. The GAPDH blot demonstrates that there was no change in the expression of total ERK1/2, total Akt1/2, and total STAT-3 in the treated group with respect to control group. Values are means ± SEM (^∗^*p* ≤ 0.05, ^∗∗^*p* ≤ 0.001) (*n* = 15 each group).

Since fingolimod pre-treatment caused a profound reduction in apoptosis of myocardial tissue, activation of pro-survival pathways was analyzed by measuring the phosphorylation state of the pro-survival Erk, Akt, and STAT3. As shown in **Figure 6**, pre-treatment with fingolimod resulted in a significant increase in phosphorylation of Erk42/44, Akt1/2, and STAT3.

### Oxidative Stress

**FIGURE 7:**
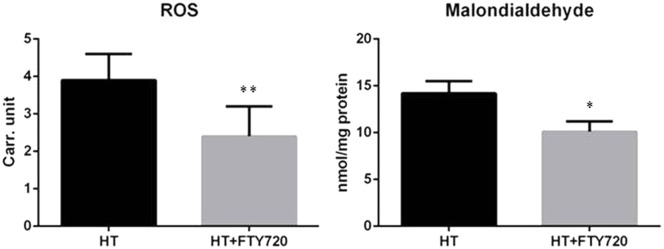
Oxidative stress comparison of oxidative stress in heterotopic transplantation model with and without fingolimod treatment (*n* = 15 each group). ROS, reactive oxygen species; HT, heterotopic transplantation; FTY720, fingolimod; Carr. unit. Carratelli unit (^∗∗^*p* ≤ 0.001, ^∗^*p* ≤ 0.05).

Uncontrolled production of reactive oxygen species (ROS) is one of the major causes inducing apoptosis in cardiac tissue after R. In order to evaluate whether fingolimod-mediated cardiac protection is due to its effect on cardiac ROS generation, levels of ROS and aldehydes (lipid peroxidation derivatives) were analyzed in the frozen perfused myocardial sample. As shown in **Figure 7**, levels of ROS and malondialdehyde were significantly reduced in cardiac tissues pre-treated with fingolimod.

## Discussion

In this study, we investigated cardioprotective and anti-fibrotic effect of pharmacological preconditioning during prolonged organ preservation using an *in vivo* heart transplantation model. We found that fingolimod, a S1PR modulator, efficiently works as ischemic preconditioning agent. Our findings suggested that fingolimod plays an important role in the reduction of apoptosis, inflammation, oxidative stress, and cardiac fibrosis. To the best of our knowledge, the present study is first to investigate cardioprotective and anti-fibrotic role of fingolimod in preclinical heart transplantation model.

Fingolimod is one of the FDA approved treatment option in prevention of multiple sclerosis relapse (Bergvall et al., 2014; Totaro et al., 2015). In addition to its immune-modulating effect, fingolimod has a number of additional useful actions, including anti-inflammatory, anti-apoptotic, anti-oxidative, and anti-nitrative stress (Behjati et al., 2014). These properties are predicted to improve the myocardial insult related to I/R during heart transplantation. Previous studies have reported that pre-treatment with S1PR modulator protected myocardium from I/R injury (Li et al., 2016). In the present study, we show that fingolimod treatment remarkably reduced myocardial fibrosis.

The role of fingolimod in cytoprotection in different organs has been reported including pancreatic transplantation in diabetics (Tchorsh-Yutsis et al., 2009), fingolimod pre-treatment reduces I/R injury in liver transplantation (Anselmo et al., 2002), in renal transplantation (Tedesco-Silva et al., 2006), and against acute ischemic stroke (Zhu et al., 2015). These all models are suggestive for cytoprotective role of fingolimod in I/R injury.

Reperfusion after transient I in myocardium leads to cardiomyocytes apoptosis and cardiac dysfunction (Khan et al., 2006; Oh et al., 2013). TUNEL assay is the gold standard method (Darzynkiewicz et al., 2008) to measure the extent of apoptosis. Consistent with previous results, the transplanted hearts treated with fingolimod 15 min before explant expressed a lower frequency of TUNEL positive nuclei compared to control. This indicates the cardioprotective role of fingolimod by activating anti-apoptotic cascade.

In order to investigate the possible mechanisms of cardio-protective effects of fingolimod, its effects on activation of survival pathways were analyzed. Activation of the RISK and SAFE pathways by fingolimod has previously been reported (Lecour et al., 2002; Zhang et al., 2007). The RISK (Akt1/2, Erk1/2, and GSK 3β) and SAFE pathways (JAK and STAT3) are the main sources for regulation of apoptotic pathways because of control on mitochondrial permeability transition pore (Heusch et al., 2011; Heusch, 2013, 2015). Consistent with previous findings, activation of RISK and SAFE signaling pathways was observed, followed by decreased level of apoptosis in the treated group as compared to control. The inhibition of pro-apoptotic protein Bax enhanced immunostaining for anti-apoptotic protein Bcl-2 was found after 24 h of reperfusion in heterotopic transplanted heart tissue.

In this study, administration of fingolimod demonstrated inhibition of apoptosis by inhibiting inflammation and oxidative stress. In our investigations, on molecular and protein level both cardioprotection have been observed. According to previous reports, S1P and its agonist has important role in reduction of inflammatory mediators in I/R injury (Stone et al., 2015). Different models have been tested for immunosuppression including the porcine model of I/R, and spontaneous obstructive coronary atherosclerosis murine model showed the better myocardial protection and decrease inflammatory markers in the fingolimod-treated group. We measured inflammatory response in blood and heart tissue. In blood, we found the reduction of neutrophils and lymphocytes. Santos-Gallego et al. (2016) found improved myocardial salvage in animals using fingolimod and suggested an immunomodulatory role of fingolimod by activation of S1PRs. The inflammatory mechanism is one of the key factors in I/R injury. The ICAM-1, IL-6, and TNF-α contribute in as a pro-inflammatory cytokines to develop myocardial damage (Bonney et al., 2013; Li et al., 2013; Markowski et al., 2013). The correlation between anti-inflammatory effects of fingolimod with reduction in fibrosis is evident in our experiment.

It is well established that I/R causes myocardial injury due to oxidative stress (Ferrari et al., 2004; Ansley and Wang, 2013). Oxidative stress is also a major factor involved in apoptosis. Treatment with fingolimod reduces apoptosis in the I/R model (Chamorro et al., 2016; Santos-Gallego et al., 2016). This reduction in apoptosis is mediated via both RISK and SAFE pathways (Santos-Gallego et al., 2016). In our transplantation experiments, decreased level of malondialdehyde and ROS in the fingolimod-treated group was observed suggesting the reduction in oxidative stress is one of the cardioprotective mechanisms of S1PR modulators.

Myocardial I/R also produces nitric oxide synthase that releases nitric oxide which reacts with ROS as a result, forms toxic peroxynitrite leading to necrosis and apoptosis (Nour et al., 2013). Fingolimod treatment partially attenuates nitrative stress in transplanted myocardium (Colombo et al., 2014). Present findings suggest fingolimod can be efficiently used as a preconditioning agent to improve myocardial salvage.

Together, all these results are suggestive of the myocardial protective role having reduced fibrosis of fingolimod in global I–R. Fingolimod is the only available FDA-approved agent, containing S1PR agonist for prevention of multiple sclerosis relapses. According to our recent study on characterization and expression of S1PRs in human and rat (Ahmed et al., 2017) indicates potential translation of current study into clinical trials.

## Conclusion

In conclusion, our study supports the cardioprotective role of S1PR modulator fingolimod for reduction in apoptosis, inflammation, oxidative stress, and fibrosis in an experimental model of heterotopic HT. This study provides insight for activation of cellular signaling pathways including RISK and SAFE pathways following significantly reduced cardiac fibrosis in long-term R.

## Author Contributions

NA, AR, GsF, GdF, and GL participated in research design; NA, DL, LSB, MG, and MA conducted experiments; AR and CC contributed new reagents; NA, NM, and MG performed data analysis; and NA, AR, and NM writing manuscript.

## Conflict of Interest Statement

The authors declare that the research was conducted in the absence of any commercial or financial relationships that could be construed as a potential conflict of interest. The reviewer LM and handling Editor declared their shared affiliation.
